# Influenza “Trains” the Host for Enhanced Susceptibility to Secondary Bacterial Infection

**DOI:** 10.1128/mBio.00810-19

**Published:** 2019-05-07

**Authors:** Kari Ann Shirey, Darren J. Perkins, Wendy Lai, Wei Zhang, Lurds R. Fernando, Fabian Gusovsky, Jorge C. G. Blanco, Stefanie N. Vogel

**Affiliations:** a Department of Microbiology and Immunology, University of Maryland School of Medicine, Baltimore, Maryland, USA; b Sigmovir Biosystems, Inc., Rockville, Maryland, USA; c Eisai Inc., Andover, Massachusetts, USA; National Institute of Allergy and Infectious Diseases; Cedars-Sinai Medical Center; Children's Hospital Boston

**Keywords:** IFN-β, MRSA, *Streptococcus pneumoniae*, TLR4, cotton rats, influenza, macrophage training, secondary bacterial infection

## Abstract

We previously reported that the Toll-like receptor 4 (TLR4) antagonist Eritoran blocks acute lung injury (ALI) therapeutically in mouse and cotton rat models of influenza. However, secondary (2°) bacterial infection following influenza virus infection is associated with excess morbidity and mortality. Wild-type (WT) mice infected with mouse-adapted influenza A/Puerto Rico/8/34 virus (PR8) and, 7 days later, with Streptococcus pneumoniae serotype 3 (*Sp3*) exhibited significantly enhanced lung pathology and lethality that was reversed by Eritoran therapy after PR8 infection but before *Sp3* infection. Cotton rats infected with nonadapted pH1N1 influenza virus and then superinfected with methicillin-resistant Staphylococcus aureus also exhibited increased lung pathology and serum high-mobility-group box 1 (HMGB1) levels, both of which were blunted by Eritoran therapy. In mice, PR8 infection suppressed *Sp3*-induced CXCL1 and CXCL2 mRNA, reducing neutrophil infiltration and increasing the bacterial burden, all of which were reversed by Eritoran treatment. While beta interferon (IFN-β)-deficient (IFN-β^−/−^) mice are highly susceptible to PR8, they exhibited delayed death upon *Sp3* superinfection, indicating that while IFN-β was protective against influenza, it negatively impacted the host response to *Sp3*. IFN-β-treated WT macrophages selectively suppressed *Sp3*-induced CXCL1/CXCL2 transcriptionally, as evidenced by reduced recruitment of RNA polymerase II to the CXCL1 promoter. Thus, influenza establishes a “trained” state of immunosuppression toward 2° bacterial infection, in part through the potent induction of IFN-β and its downstream transcriptional regulation of chemokines, an effect reversed by Eritoran.

## Abstract (precis)

Enhanced susceptibility to 2° bacterial infections following infection with influenza virus is a global health concern that accounts for many hospitalizations and deaths, particularly during pandemics. The complexity of the impaired host immune response during 2° bacterial infection has been widely studied. Both type I IFN and neutrophil dysfunction through decreased chemokine production have been implicated as mechanisms underlying enhanced susceptibility to 2° bacterial infections. Our findings support the conclusion that selective suppression of CXCL1/CXCL2 represents an IFN-β-mediated “training” of the macrophage transcriptional response to TLR2 agonists and that blocking of TLR4 therapeutically with Eritoran after influenza virus infection reverses this suppression by blunting influenza-induced IFN-β.

## INTRODUCTION

Influenza is a major global health concern with seasonal outbreaks and pandemics that result in significant morbidity and mortality (1, 2). The 2017-2018 influenza season showed significant increases in hospitalizations confirmed to be due to influenza for both adults and children in the United States alone (3). While vaccination provides significant protection, the ability to predict the specific influenza virus strains to be incorporated into the following year’s vaccine sometimes fails (4, 5), which can lead to a virus-vaccine mismatch and reduced vaccine efficacy (3). In addition to having to treat patients early in infection, increasing resistance to neuraminidase (NA) inhibitors (oseltamivir, zanamivir) and M2 channel inhibitors (amantadine, rimantadine) has limited the utility of antiviral drugs (6, 7). Thus, host-directed therapeutics represent an alternate approach to treating severe influenza virus infection.

In a review by Abramson and Mills (8), it was stated that one of the earliest documented reports that viral infection could lead to enhanced susceptibility to bacterial infection was published in 1908. Bacterial coinfection was associated with nearly all influenza-attributed deaths in the 1918 pandemic (9) and up to 34% of 2009 pandemic influenza A virus (H1N1) infections (10). Bacterial coinfection commonly occurs within the first 6 days of influenza virus infection, with Streptococcus pneumoniae and Staphylococcus aureus being most commonly isolated (11). Given our previous studies showing that the Toll-like receptor 4 (TLR4) antagonist Eritoran (E5564), as well as other structurally unrelated TLR4 antagonists, blocked influenza-induced acute lung injury (ALI) in wild-type (WT) mice and in cotton rats (12–15), we sought to determine if such treatment would also mitigate the increased susceptibility of the host to secondary (2°) bacterial infection.

## RESULTS

### E5564 protects mice from secondary bacterial infection after primary influenza virus infection.

**FIG 1:**
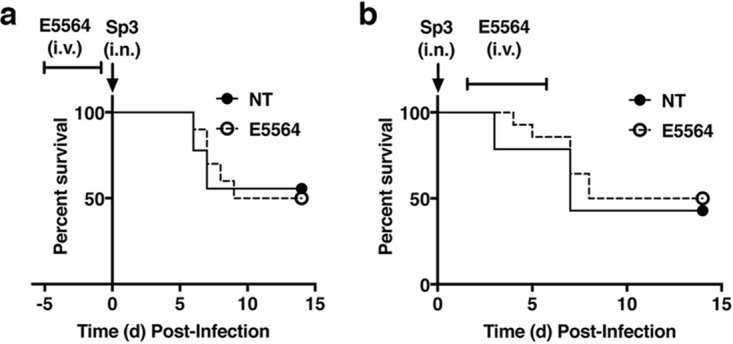
Eritoran (E5564) treatment does not affect survival during *Sp3* infection. (a) WT C57BL/6J mice were either left untreated (NT) or treated with E5564 (200 μg/mouse i.v.) once daily for 5 consecutive days (days −5 to −1) prior to infection with an ∼LD_40_ of *Sp3* (∼1,500 CFU/mouse i.n.) on day (d) 0. Mice were monitored daily for survival for 14 days post-*Sp3* infection. (b) WT C57BL/6J mice were infected with an ∼LD_40_ of *Sp3* (∼1,500 CFU/mouse i.n.) on day 0. Mice were either left untreated or treated with E5564 (200 μg/mouse i.v.) once daily for 5 consecutive days starting on day 2 postinfection (days 2 to 6). Mice were monitored daily for survival for 14 days post-*Sp3* infection. Results represent combined data from 2 separate assays (4 to 5 mice/treatment group/experiment).

Initially, we assessed the efficacy of prophylactic or therapeutic Eritoran (E5564) treatment in mice infected with Streptococcus pneumoniae serotype 3 (*Sp3*). WT C57BL/6J mice were either left untreated (NT) or treated once daily prophylactically (days −5 to −1) or therapeutically (days 2 to 6) with Eritoran. On day 0, mice were infected with *Sp3* (∼1 40% lethal dose [LD_40_]). Neither Eritoran prophylaxis nor therapy affected the survival of *Sp3*-infected mice (Fig. 1a and b).

**FIG 2:**
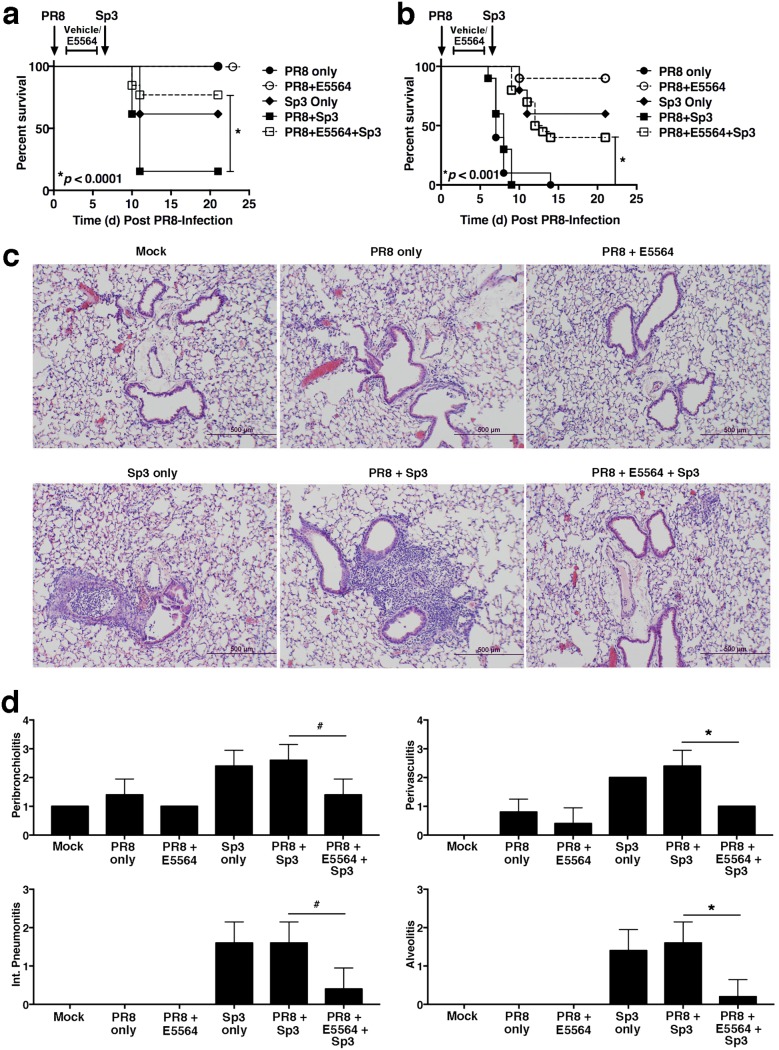
E5564 protects mice and mitigates ALI from secondary bacterial infection after primary influenza virus infection. (A) WT C57BL/6J mice were infected on day 0 with a nonlethal dose of influenza A/Puerto Rico/8/34 virus (PR8; ∼1,000 TCID_50_ i.n.). Mice were treated with E5564 (200 μg/mouse i.v.) daily on days 2 to 6 post-PR8 challenge. On day 7, mice were challenged with an ∼LD_40_ of *Sp3* (∼1,500 CFU). Mice were monitored for survival through 21 days post-PR8 challenge (14 days post-*Sp3* challenge). Data represent the combined results from 2 separate assays (6 to 7 mice/treatment group/experiment). (B) WT C57BL/6J mice were infected on day 0 with a lethal dose of PR8 (∼7,500 TCID_50_ i.n.). Mice were treated with E5564 as described in the legend to panel A. On day 7, mice were challenged with an ∼LD_40_ of *Sp3*. Mice were monitored for survival as described in the legend to panel A. Data represent the combined results from 2 separate assays (10 mice/treatment group/experiment). (C and D) WT C57BL/6J mice were infected on day 0 with a nonlethal dose of PR8 (∼1,000 TCID_50_ i.n.). Mice were treated with E5564 (200 μg/mouse i.v.) from days 2 to 6 post-PR8 challenge. On day 7, mice were challenged with an ∼LD_40_ of *Sp3* (∼1,500 CFU). At 2 days post-*Sp3* infection (9 days post-PR8 infection), mice were euthanized and the lungs were extracted for H&E staining (C) and histopathology scoring (D). Representative sections are shown in panel C. Int., interstitial. Data are for 5 mice/treatment group/experiment from two separate experiments. #, *P < *0.05; *, *P < *0.01.

We developed a model of secondary bacterial infection that elicits enhanced mortality (16, 17) to test our hypothesis that Eritoran therapy would ameliorate the enhanced susceptibility associated with bacterial infection postinfluenza. WT mice were infected on day 0 with a nonlethal dose of influenza A/Puerto Rico/8/34 virus (PR8) (∼1,000 50% tissue culture infective doses [TCID_50_]), followed on day 7 by *Sp3* infection (∼1 LD_40_). Figure 2a shows that all mice that were infected on day 0 with this nonlethal dose of PR8 survived. Another set of control mice that were mock infected on day 0 and then infected with *Sp3* on day 7 exhibited ∼40% lethality, as expected. When mice were PR8 infected on day 0, vehicle treated on days 2 to 6, and then infected with *Sp3* on day 7, there was a highly significant increase in lethality (∼90%), confirming that this is a robust model of influenza-enhanced susceptibility to 2° bacterial infection.

To determine if Eritoran (E5564) treatment of influenza virus-infected mice would affect susceptibility to 2° bacterial infection, mice were infected with PR8 on day 0, treated with vehicle or Eritoran on days 2 to 6, and then superinfected with *Sp3* on day 7. As expected, Eritoran treatment did not affect the survival of the PR8-infected mice (in the absence of *Sp3* infection) (Fig. 2A, dashed line, open circle). However, mice treated with Eritoran on days 2 to 6 after PR8 infection showed significantly enhanced protection from *Sp3*-induced lethality (from ∼90% to ∼20% lethality; *P* < 0.0001).

We repeated these experiments using a more stringent model of PR8-induced lethality. Mice were infected with PR8 at a dose that typically kills ∼90% of infected mice (12, 13). In these experiments, all mice infected with this dose of PR8 succumbed to infection within 2 weeks (Fig. 2B, solid line, closed circles). Eritoran treatment of a similarly PR8-infected group resulted in ∼90% survival, as previously reported (Fig. 2B, dashed line, open circles) (12, 13). *Sp3*-infected control mice (no PR8 infection, no treatment; Fig. 2B, solid line, diamond) had ∼40% lethality. However, mice infected with a lethal dose of PR8, treated with Eritoran, and then superinfected with *Sp3* showed a highly significant degree of improvement (*P* < 0.001), from 0% survival by day 9 (Fig. 2B, solid line, solid squares) to ∼50% (Fig. 2B, dashed line, open squares). These data extend our findings presented in Fig. 2A by showing that Eritoran therapy mitigates the enhanced lethality that occurs after lethal influenza virus challenge followed by *Sp3* superinfection.

### Treatment of mice with Eritoran after PR8 infection and prior to *Sp3* infection mitigates lung pathology.

To examine the effect of Eritoran treatment on lung inflammatory responses following PR8 and/or *Sp3* infection, mice were infected as described above in the assay whose results are presented in Fig. 2A. At 2 days after *Sp3* infection (day 9 postinfection [p.i.]), all mice were euthanized. Mock-infected lungs had a normal lung architecture with clear airways, an intact airway epithelium, and no cell infiltrates (Fig. 2C, top left). Mice infected with a nonlethal PR8 dose alone showed minimal lung pathology (mild peribronchiolar and perivascular infiltrates) that was alleviated by Eritoran treatment (Fig. 2C, top middle and top right, respectively). Mice infected with *Sp3* alone showed lung damage that included bronchial plugs filled with neutrophils, marked peribronchiolar and perivascular neutrophilic inflammation, and patches of pneumonia consisting of both interstitial and alveolar neutrophilic infiltrates (Fig. 2C, bottom left). The lungs of mice infected with PR8, vehicle treated, and then *Sp3* infected showed a worsened histopathology, i.e., strong peribronchiolitis and alveolitis with several patches of pneumonia and inflammatory mononuclear infiltrates (Fig. 2C, bottom middle). However, lung sections of PR8-infected, Eritoran-treated mice that were subsequently infected with *Sp3* exhibited a significantly diminished lung pathology (Fig. 2C, bottom right) compared with that of mice infected with PR8 followed by *Sp3* infection or mice infected with *Sp3* only. These observations are supported by histological scores determined in a blind manner (Fig. 2D).

In contrast to mice, cotton rats (Sigmodon hispidus) are susceptible to nonadapted human strains of influenza virus (18) and can also be protected from influenza-induced ALI by Eritoran therapy (12). Therefore, using the same protocol used for the mice, we tested the efficacy of Eritoran therapy in cotton rats infected with a nonadapted human influenza virus strain, CDC A/California/7/2009 (pH1N1) virus, followed by infection with methicillin-resistant Staphylococcus aureus (MRSA), shown to be associated with an increased risk of severe disease in children and adults after influenza virus infection (19–21). Cotton rats were infected with influenza virus on day 0, followed by treatment with saline or Eritoran for 5 consecutive days starting on day 2 post-influenza virus challenge. On day 7 post-influenza virus challenge, animals were infected with MRSA. On day 6 post-MRSA infection (day 13 post-influenza virus challenge), lungs were collected for histopathology and scored in a blind manner. Mock-infected animals had normal lung histology (see Fig. S1a and d in the supplemental material), while those infected with pH1N1 and then secondarily infected with MRSA showed severe pathology, with inflammatory infiltrates throughout the lungs, peribronchiolitis, and perivasculitis being seen (Fig. S1b and e). Lungs from cotton rats treated with Eritoran showed a reduced accumulation of inflammatory cells surrounding the airways and vasculature (Fig. S1c and f).

10.1128/mBio.00810-19.1 FIG S1 Eritoran treatment reduces pH1N1- and MRSA-induced lung pathology and HMGB1 levels in cotton rats. Cotton rats (5 to 15 animals/group) were infected with influenza virus (pH1N1 at 2.15 × 10^6^ PFU/ml i.n.) on day 0, followed by treatment with E5564 or saline daily for 6 successive days (days 1 to 6, i.v., 9.33 μg/μl, 200 μl/animal). On day 7 post-influenza virus infection, half of the animals in each group were infected with MRSA (strain MBT 5040, i.n., 10% transmittance). Lungs were collected on day 6 post-MRSA challenge (day 13 post-influenza virus infection) for histopathology analysis. (a and d) Representative sections from mock-infected animals; (b and e) representative sections for influenza virus pH1N1- and MRSA-infected, saline-treated animals; (c and f) representative sections of influenza virus pH1N1- and MRSA-infected, E5564-treated animals. Bars, 1.0 mm (a to c) and 500 μm (d to f). (g) Serum was collected from cotton rats for histology, and HMGB1 protein levels were measured by ELISA. The data presented are the means ± SEM (*n* = 5/8 animals/treatment group). ***, *P < *0.0001; ****, *P < *0.00001. Download FIG S1, JPG file, 1.2 MB. Copyright © 2019 Shirey et al. 2019 Shirey et al. This content is distributed under the terms of the Creative Commons Attribution 4.0 International license.

While influenza virus does not express TLR4 pathogen-associated molecular patterns (PAMPs), infection elicits increased levels of a host-derived damage-associated molecular pattern (DAMP), high-mobility-group box 1 (HMGB1), shown previously to be a TLR4 agonist (13, 22). Enhanced HMGB1 levels correlate with the severity of ALI induced by nonadapted influenza virus strains in the cotton rat model (23), and administration of an HMGB1 small-molecule inhibitor to PR8-infected mice significantly enhanced survival, comparable to the findings seen with Eritoran treatment (13). Serum levels of HMGB1 were significantly lower in cotton rats that received Eritoran before 2° challenge with MRSA (Fig. S1g).

Antagonizing TLR4 with Eritoran and other TLR4 antagonists blunts the proinflammatory gene expression induced by influenza virus infection in the lung (12, 14, 15). Eritoran treatment of mice infected with a sublethal dose of PR8 blunted expression of genes encoding interleukin-1β (IL-1β), tumor necrosis factor alpha (TNF-α), and COX2, measured on day 9 postinfection (p.i.) (Fig. S2, PR8 + E5564). However, by day 9, the level of beta interferon (IFN-β) mRNA in mice infected with PR8 had largely returned to baseline after peaking on days 4 to 6 (12). In mice infected with *Sp3* alone or infected with *Sp3* following PR8 infection, there was a strong increase in the expression of all 4 cytokine genes measured in the lung on day 9. Importantly, those that received Eritoran treatment after PR8 infection but prior to *Sp3* superinfection showed a significant decrease (*P* < 0.05) in both COX2 and IFN-β mRNA expression and a nonsignificant trend toward decreased IL-1β and TNF-α mRNA expression (Fig. S2).

10.1128/mBio.00810-19.2 FIG S2 Effect of Eritoran on PR8- and/or *Sp3*-induced proinflammatory gene expression. WT C57BL/6J mice were infected on day 0 with a nonlethal dose of PR8 (∼1,000 TCID_50_ i.n.). Mice were left untreated or treated with E5564 (200 μg/mouse; i.v.) from days 2 to 6 post-PR8 challenge. On day 7, mice were challenged with an ∼LD_40_ of *Sp3* (∼1,500 CFU/mouse i.n.). At 2 days post-*Sp3* infection (9 days post-PR8 infection), mice were euthanized and lungs were extracted for mRNA analysis. The data shown are from 2 separate experiments and are for 5 mice/group/experiment. #, *P < *0.05. Download FIG S2, JPG file, 0.1 MB. Copyright © 2019 Shirey et al. 2019 Shirey et al. This content is distributed under the terms of the Creative Commons Attribution 4.0 International license.

### Eritoran treatment restores neutrophil function.

**FIG 3:**
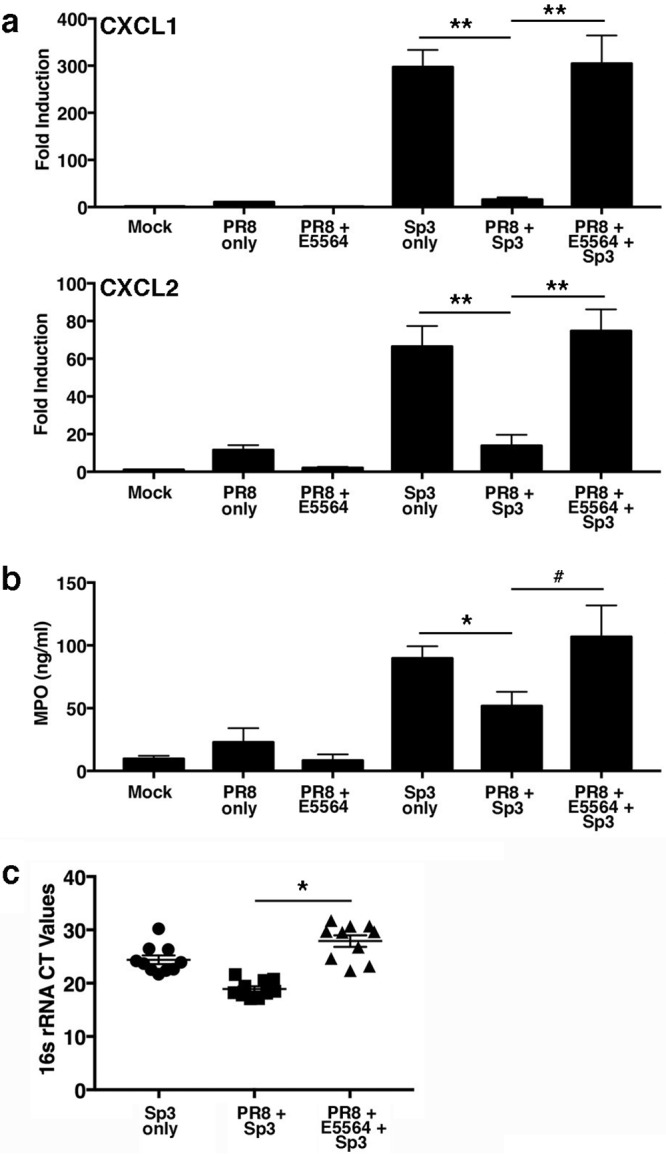
E5564 treatment reverses suppression of neutrophil chemokine gene expression in the lungs of PR8- and *Sp3*-infected mice. WT C57BL/6J mice were infected on day 0 with a nonlethal dose of PR8 (∼1,000 TCID_50_ i.n.). Mice were treated with E5564 (200 μg/mouse i.v.) from days 2 to 6 post-PR8 challenge. On day 7, mice were challenged with an ∼LD_40_ of *Sp3* (∼1,500 CFU i.n.). At 2 days post-*Sp3* infection (9 days post-PR8 infection), mice were euthanized and lungs were extracted for determination of mRNA gene expression (a), myeloperoxidase (MPO) activity (b), and 16S rRNA expression for *Sp3* (c). Data are derived from 2 separate experiments and are for 5 mice/treatment group/experiment. #, *P < *0.05; *, *P < *0.01; **, *P < *0.001.

Influenza virus infection causes neutrophil infiltration into the lungs (Fig. 2 and Fig. S1) (24). Ishikawa et al. reported that susceptibility to 2° bacterial infection after influenza was secondary to neutrophil dysfunction when mice were infected with Pseudomonas aeruginosa 4 days after PR8 infection due to a decrease in granulocyte colony-stimulating factor activity (25), accompanied by decreased myeloperoxidase (MPO) activity, in the bronchoalveolar lavage fluids of mice infected with PR8 and then P. aeruginosa (25). Robinson et al. reported that neutrophil chemokine expression was depressed after influenza virus infection and proposed that this contributed to increased susceptibility to 2° Staphylococcus aureus infection (26). PR8-infected mice exhibited increased lung CXCL1 (keratinocyte-derived chemokine [KC]) mRNA expression on days 4 to 6 after challenge that was inhibited by Eritoran treatment (12). Thus, we sought to determine if Eritoran therapy after PR8 infection would modulate neutrophil chemokine expression in our model of 2° bacterial pneumonia. Mice infected with a nonlethal dose of PR8 induced an ∼10- and 12-fold increase in gene expression of the neutrophil chemokines CXCL1 (KC) and CXCL2 (macrophage inflammatory protein 2α), respectively, at day 9, with Eritoran treatment decreasing their expression (Fig. 3a). Mice infected with *Sp3* alone showed much higher levels of both CXCL1 (∼30-fold) and CXCL2 (∼4-fold) mRNA than mice that were infected with influenza virus, followed by *Sp3*. These data indicate that PR8 suppresses *Sp3*-induced chemokine gene expression. Surprisingly, treatment of PR8-infected mice with Eritoran prior to *Sp3* infection reversed this suppression (Fig. 3a). These observations were paralleled by the MPO activity in lung homogenates, an indirect measure of neutrophil infiltration (Fig. 3b).

*Sp3* 16S rRNA was measured in these samples by quantitative PCR (qPCR) to quantify the bacterial load in the lungs of *Sp3*-challenged animals. *Sp3* levels inversely correlated with MPO activity. Figure 3c shows that mice infected with *Sp3* after influenza virus infection had a lower bacterial load (a higher cycle threshold [*C_T_*] value) than mice infected with *Sp3* alone. Importantly, mice that were infected with PR8, treated with Eritoran, and then infected with *Sp3* had significantly less lung *Sp3* 16S rRNA than mice infected with PR8, followed by vehicle treatment and then by *Sp3* infection (*P* < 0.01). These data support our survival data (Fig. 1) and indicate that Eritoran therapy after PR8 infection, but prior to *Sp3* infection, improves survival by limiting the proinflammatory response while enabling neutrophil recruitment to the lung, which, in turn, controls bacterial replication.

**FIG 4:**
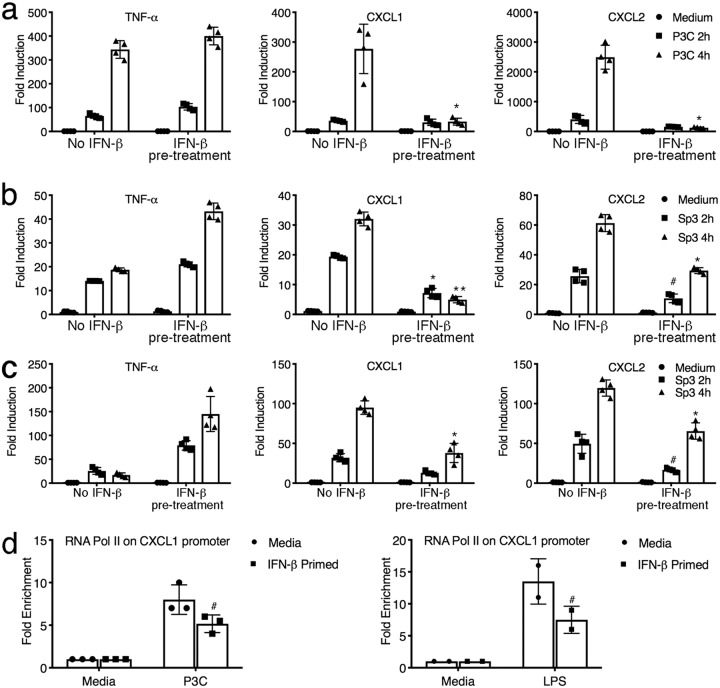
IFN-β suppresses transcriptional induction of chemokine gene expression *in vitro.* (a) WT C57BL/6J macrophages were pretreated for 1 h with medium alone or IFN-β (100 U/ml). Macrophages were stimulated with the TLR2 agonist P3C (250 ng/ml) for 2 h or 4 h. RNA was harvested, and mRNA gene expression was analyzed by qRT-PCR. Data are shown as the mean ± SD. *, *P < *0.01. (b) MH-S cells were pretreated as described in the legend to panel a and stimulated with *Sp3* (MOI = 1), and gene expression was analyzed by qRT-PCR at the indicated times. Data are representative of those from two independent experiments. Data are shown as the mean ± SD. #, *P < *0.05; *, *P < *0.01; **, *P < *0.001. (c) WT C57BL/6J macrophages were pretreated and subsequently treated with *Sp3* as described in the legend to panel b, and gene expression was analyzed. Data are shown as the mean ± SD. #, *P* < 0.05; *, *P* < 0.01. (d) Peritoneal macrophages were primed with 100 U/ml IFN-β prior to stimulation with either P3C (100 ng/ml) or LPS (100 ng/ml) for 3 h. RNA Pol II recruitment to the CXCL1 promoter was quantified by a chromatin immunoprecipitation assay using an antibody directed against mouse RNA Pol II. #, *P < *0.05. Data are representative of those from two independent experiments. The data shown are the mean ± SD.

Influenza virus is a potent inducer of type I IFNs (12, 27). Perkins et al. reported that macrophages selectively suppressed innate immune signaling when treated with IFN-β prior to stimulation with either the TLR4 agonist lipopolysaccharide (LPS) or the synthetic TLR2 agonist Pam3CysSerLys4 (P3C) (28). Since *Sp3* is predominantly a TLR2-dependent pathogen (29–31), we assessed the effect of IFN-β pretreatment on TLR2-mediated CXCL1 and CXCL2 induction. WT peritoneal macrophages were stimulated with IFN-β for 1 h, followed by stimulation with P3C for 2 or 4 h. While TNF-α mRNA was not affected by IFN-β pretreatment, we observed a selective repression of gene expression of CXCL1 and CXCL2 mRNA (Fig. 4a; *P < *0.01). Similarly, when the murine alveolar macrophage cell line MH-S was treated with IFN-β, followed by stimulation with *Sp3* for 2 or 4 h, *Sp3*-inducible CXCL1 and CXCL2 mRNA expression was significantly lower (Fig. 4b; *P < *0.01 and *P < *0.05, respectively), consistent with the data in Fig. 4a, whereas TNF-α mRNA expression was increased slightly (Fig. 4b). The same trend shown in Fig. 4b was observed when peritoneal macrophages were stimulated with *Sp3* (Fig. 4c). Thus, IFN-β selectively alters macrophage sensitivity to subsequent TLR2 stimulation by either synthetic or infectious agonists at the level of steady-state mRNA, which parallels our observations of the effect of influenza virus infection and *Sp3* superinfection *in vivo*.

Recent studies have demonstrated that prior infections can selectively alter the expression of specific innate immune genes, often by epigenetic changes (32, 33), such that the response to subsequent infection is altered. This has been referred to as “training” of the innate immune response (32). To further extend our findings, we hypothesized that influenza-induced IFN-β trains the macrophage response to *Sp3*, such that the induction of neutrophil chemokine mRNA expression is transcriptionally modified. That the observed inhibition of CXCL1 mRNA is a form of macrophage training induced by IFN-β is supported by decreased RNA polymerase II (Pol II) recruitment to the *Cxcl1* promoter in macrophages secondarily stimulated with P3C or LPS (Fig. 4d).

We next compared the responses of WT mice with those of IFN-β^−/−^ mice to establish the role of influenza-induced IFN-β in 2° bacterial infection. IFN-β^−/−^ mice are considerably more sensitive to PR8 infection than WT mice (27). Therefore, we first determined a nonlethal dose of PR8 in IFN-β^−/−^ mice (500 TCID_50_). The survival of PR8-infected mice was only ∼10% when either WT or IFN-β^−/−^ mice were infected with this dose of PR8 and superinfected with *Sp3* (Fig. S3). While a similar degree of sensitization occurred in the PR8-infected, *Sp3*-infected WT and IFN-β-deficient mice, there was an ∼4-day delay in the mean time to death in the IFN-β^−/−^ mice (*P* = 0.008), supporting the notion that type I IFNs, such as IFN-β, are detrimental to the host during 2° bacterial infection.

10.1128/mBio.00810-19.3 FIG S3 IFN-β deficiency significantly extends the mean time to death in secondary bacterial infection. WT C57BL/6J and IFN-β^−/−^ mice were infected on day 0 with a dose of PR8 that was determined not to be lethal in IFN-β^−/−^ mice (∼500 TCID_50_ i.n.). Mice were treated with E5564 (200 μg/mouse i.v.) from days 2 to 6 post-PR8 challenge. On day 7, mice were challenged with an ∼LD_40_ of *Sp3* (∼1,500 CFU). Mice were monitored for survival up through 21 days post-PR8 challenge and 14 days post-*Sp3* challenge. The data are the combined results from three separate assays (6 to 7 mice/treatment group/experiment). Download FIG S3, JPG file, 0.2 MB. Copyright © 2019 Shirey et al. 2019 Shirey et al. This content is distributed under the terms of the Creative Commons Attribution 4.0 International license.

## DISCUSSION

Influenza virus causes infection in the respiratory tract that can lead to a robust proinflammatory response but that can also result in the host immune response becoming compromised, leading to increased susceptibility to secondary infections (9, 10). Secondary bacterial infections following primary infections with influenza virus are a frequent complication resulting in the majority of deaths during pandemics (9). These bacterial infections usually begin within 7 days of the influenza virus infection (11); however, studies have shown that there may still be an increased risk of a 2° bacterial infection even after viral clearance (34, 35).

While studies done by multiple groups have suggested the complexity of the immune response during a 2° bacterial infection (36, 37), our goal was to understand if and how Eritoran treatment of primary influenza virus infection might affect the response to bacterial infection. We have previously shown that therapeutic treatment with Eritoran during lethal influenza virus infection significantly protects both mice and cotton rats by blunting the expression of multiple cytokines and chemokines (12). However, whether this would then render the host more susceptible to a 2° bacterial infection was unknown. We first assessed the efficacy of Eritoran treatment of a low-dose viral infection prior to exposure to *Sp3*. Our findings indicate that even a sublethal exposure to influenza virus greatly enhances susceptibility to bacterial pneumonia, consistent with the findings of other studies. More importantly, our findings show that Eritoran treatment of PR8-infected mice can, indeed, blunt the enhanced susceptibility to bacterial superinfection. Moreover, protection was observed even when a lethal dose of influenza virus was used to infect mice prior to *Sp3* infection.

Neutrophil function has been shown to be impaired following influenza virus infection (38–43). Shahangian et al. reported that a decrease in the chemokines CXCL1 and CXCL2 during coinfection with *Sp3* rendered the mice more sensitive and that this was linked to type I IFNs by showing that IFN-α/β receptor knockout mice had higher numbers of neutrophils recruited to the lung in response to 2° *Sp3* infection (43). To further illustrate the importance of these chemokines during 2° bacterial infection, the authors exogenously treated WT mice with CXCL1 and CXCL2 at the time that the mice were infected with *Sp3* after primary influenza virus infection. Mice treated with the chemokines exhibited increased MPO levels and a decreased bacterial burden (43). Consistent with these findings, our data show that the decrease in CXCL1 and CXCL2 and neutrophil MPO activity during 2° bacterial infection with *Sp3* is reversed when the mice are treated with Eritoran prior to bacterial infection. Mechanistically, we show that the type I IFN-mediated decrease in chemokine induction is secondary to the decreased recruitment of RNA Pol II to the chemokine promoter. We previously reported that treatment of PR8-infected mice with Eritoran significantly blunted type I IFN induction (12), and these findings were confirmed here. Collectively, our data support the hypothesis that the reduced levels of IFN-β in Eritoran-treated mice prior to 2° S. pneumoniae infection preclude training of the macrophages, possibly by preventing IFN-inducible epigenetic changes, such that neutrophil chemokines are able to be induced upon *Sp3* infection (Fig. 3a).

Type I IFNs upregulate a large number of genes, some of which are known to have antiviral properties (44), while others have suggested that type I IFN production during influenza virus infection suppresses the production of antimicrobial peptides that enhance susceptibility to 2° bacterial infection (45). Mechanistically, how type I IFNs suppress inflammatory and antimicrobial gene transcription is still being elucidated. One report identified promoter-specific H3K9Me3 epigenetic modifications in the CXCL1 gene caused by exposure to type I IFNs (46). This report also showed that, *in vivo*, an interferon-induced H3K9 lysine methyltransferase, Setdb2, increased susceptibility to bacterial superinfection following influenza (46). Our own *in vitro* analysis did not, however, identify a reduced capacity for IFN-β to suppress CXCL1 and CXCL2 transcription in Setdb2^−/−^ macrophages (see Fig. S4 in the supplemental material). Our data support the concept that while influenza virus sensitivity is highly IFN-β dependent, the IFN-β induced in response to influenza virus infection is highly detrimental for the host response to secondary bacterial infection. This observation also supports previous reports that mice deficient in either STAT1 or STAT2, transcription factors activated by type I IFNs, also exhibit some degree of protection from 2° bacterial infection (45, 47).

10.1128/mBio.00810-19.4 FIG S4 WT and Setdb2^−/−^ macrophages respond comparably to IFN-β-mediated suppression of chemokines. (A to C) Primary murine bone marrow-derived macrophages generated from WT and Setdb2 conditional knockout mice were pretreated for 4 h with medium alone (media) or medium supplemented with recombinant murine IFN-β (100 U/ml). Following pretreatment, macrophages were stimulated with E. coli LPS (100 ng/ml) for 18 h. Medium supernatants were harvested, and cytokine levels were quantified by ELISA. The data presented are the combined results of two independent experiments with three technical replicates. Download FIG S4, JPG file, 0.1 MB. Copyright © 2019 Shirey et al. 2019 Shirey et al. This content is distributed under the terms of the Creative Commons Attribution 4.0 International license.

This study has important clinical ramifications for the dual role of IFN-β during both primary influenza virus infection and 2° bacterial infection. IFN-β deficiency results in enhanced susceptibility to primary influenza virus infection (27) yet is protective in this model of secondary bacterial infection (Fig. 2). Although treatment of mice with the TLR4-specific inhibitor Eritoran blunts IFN-β gene and protein expression in influenza virus-infected mice, the mice survive an otherwise lethal infection (12). Earlier studies indicated that Eritoran acts by blocking the TLR4-mediated signaling induced by the influenza-induced DAMP HMGB1 (13). We have also reported that influenza virus-infected, Eritoran-treated mice survive a later challenge with influenza virus and that these mice make neutralizing antibodies, indicating that the adaptive immune response is not inhibited by Eritoran therapy (13). We speculate that influenza-induced IFN-β reprograms the macrophage transcriptional response to TLR2 agonists, such that expression of neutrophil chemokine genes is repressed, leading to the observed increased sensitivity to 2° bacterial infection. By blunting influenza-induced IFN-β induction by Eritoran, the increased sensitivity to Gram-positive bacterial PAMPs is prevented.

Collectively, the data presented herein greatly extend our earlier observations of the relevance of treating influenza-induced ALI with TLR4 antagonists (12–15) by further providing a rational therapeutic approach to ameliorate the lethal effects of the 2° bacterial pneumonia that often follows influenza virus infection. Moreover, we have provided important, new mechanistic insights by showing that Eritoran protects mice against 2° bacterial infection by restoring the otherwise suppressed neutrophil infiltration that is necessary for bacterial clearance. Our data support a model in which influenza virus infection, through induction of IFN-β and its downstream effects on transcriptional regulation, establishes a trained state of immunosuppression in macrophages that leads to more severe 2° bacterial infection.

## MATERIALS AND METHODS

### Reagents.

Eritoran (E5564) was kindly provided by Eisai, Inc. (Andover, MA). Eritoran was prepared at 2.33 mg/ml as previously described (12). A mouse myeloperoxidase (MPO) kit was purchased from Abcam (Cambridge, MA). AN HMGB1 enzyme-linked immunosorbent assay (ELISA) kit was purchased from Tecan US, Inc. (Morrisville, NC). Pam3CysSerLys4 (P3C) was purchased from InvivoGen (San Diego, CA). Escherichia coli K235 LPS was prepared as previously described (48).

### Mice and cotton rats.

Six- to 8-week-old male and female WT C57BL/6J mice were purchased (The Jackson Laboratory, Bar Harbor, ME). IFN-β^−/−^ mice (provided by Eleanor Fish, University of Toronto), backcrossed >10 generations on a C57BL/6J background, were bred in the University of Maryland, Baltimore’s accredited facility. Inbred young adult (4- to 8-week-old) cotton rats (Sigmodon hispidus) were bred at Sigmovir Biosystems, Inc. (Rockville, MD). All animal experiments were conducted with institutional IACUC approval from the University of Maryland, Baltimore, and Sigmovir Biosystems, Inc.

### Pathogens.

Mouse-adapted H1N1 influenza A/PR/8/34 virus (PR8) (ATCC, Manassas, VA) was grown in the allantoic fluid of 10-day-old embryonated chicken eggs as described previously (49) and was kindly provided by Donna Farber (Columbia University). The Streptococcus pneumoniae serotype 3 (*Sp3*) isolate (ATCC 6303; ATCC, Manassas, VA) was grown in brain heart infusion broth overnight at 37°C in 5% CO_2_ and was kindly provided by Alan Cross (University of Maryland School of Medicine). The numbers of CFU for challenges were calculated from colonies plated and grown on sheep blood agar plates.

### Virus challenge and treatment.

For survival experiments, WT mice were infected with mouse-adapted influenza virus strain PR8 (∼7,500 TCID_50_ intranasally [i.n.], 25 μl/nares). This dose was found in previous experiments to kill ∼90% of infected mice (12). At 2 days after infection, mice either received vehicle or were treated with E5564 (200 μg/mouse in 100 μl intravenously [i.v.]) daily for 5 consecutive days (day 2 until day 6). On day 7 post-influenza virus infection, groups of mice were either inoculated with saline or infected with an LD_40_ of *Sp3* (∼1,500 CFU i.n., 25 μl/nares). Mice were monitored daily for survival, weight loss, and clinical signs of illness (12) for 14 days post-*Sp3* challenge. In some experiments, mice were euthanized at day 9 post-PR8 infection (2 days post-*Sp3* challenge) to harvest lungs for analysis of gene expression, MPO activity, lung pathology, and bacterial burden. In a separate series of experiments, C57BL/6J WT mice and IFN-β^−/−^ mice were infected with mouse-adapted influenza virus strain PR8 (∼500 TCID_50_ i.n., 25 μl/nares). On day 7 post-influenza virus infection, groups of mice were either inoculated with saline or infected with an LD_40_ of *Sp3* (∼1,500 CFU i.n., 25 μl/nares). Mice were monitored daily for survival for 14 days post-*Sp3* challenge.

### Cotton rat assays.

Cotton rats (4 weeks old) were separated into groups of 5 to 15 animals each. Animals were first infected with influenza virus (pH1N1 i.n. at 2.15 × 10^6^ PFU/ml) on day 0, followed by treatment with Eritoran or saline daily for 6 successive days (days 1 to 6, i.v., 9.332 μg/μl, 200 μl/cotton rat). On day 7 after influenza virus infection, half of the animals in each group were infected with methicillin-resistant Staphylococcus aureus (MRSA; strain MBT 5040 i.n. at 10% transmittance). Each animal was weighed daily. Blood samples were collected on days 11 and 13 post-influenza virus infection for analysis of HMBG1 in serum by ELISA, animals were sacrificed on day 13 post-influenza virus infection, and the lungs were collected for histology.

### Histology.

For all histology, lungs were inflated, perfused, and fixed with 4% paraformaldehyde. Fixed sections (5 μm) of paraffin-embedded lung tissues were stained with hematoxylin and eosin (H&E). Slides were randomized, read in a blind manner, and examined for tissue damage, peribronchiolitis, perivasculitis, interstitial pneumonitis, alveolitis, and inflammatory cellular infiltration (12).

### Macrophage cell cultures and treatment.

Thioglycolate-elicited peritoneal macrophages from WT C57BL/6J mice were enriched as described previously (50), after plating in 12-well tissue culture plates (2 × 10^6^ cells/well). Macrophages were pretreated with IFN-β (100 U/ml) for 1 h and then stimulated with P3C (250 ng/ml) or *Sp3* (multiplicity of infection [MOI] = 1) for 2 h or 4 h. MH-S cells, a murine alveolar macrophage cell line, were plated in 12-well tissue culture plates (2 × 10^6^ cells/well) and stimulated with IFN-β (100 U/ml) prior to stimulation with *Sp3* (MOI = 1) for 2 h or 4 h. For bone marrow-derived macrophages (BMDM), bone marrow was cultured as previously described (51). WT and Setdb2-conditional-knockout BMDM (kindly provided by Steven L. Kunkel, University of Michigan Medical School) were treated for 4 h with medium alone or IFN-β (100 U/ml). Cells were then stimulated with E. coli LPS (100 ng/ml) and incubated for an additional 18 h. Cell supernatants were harvested, and protein levels were measured by ELISA.

### qRT-PCR.

Total RNA isolation and quantitative real-time PCR (qRT-PCR) were performed as previously described (52). The levels of mRNA for specific genes are reported as relative gene expression normalized to the mRNA levels expressed by mock-infected lungs. The levels of 16S rRNA for *Sp3* (53) are reported as the direct *C_T_* value. The sequences used to detect 16S rRNA were GGTGAGTAACGCGTAGGTAA (forward) and ACGATCCGAAAACCTTCTTC (reverse).

### Lung MPO measurements.

Approximately 50 mg of lung tissue was homogenized, and MPO levels were measured according to the manufacturer’s protocol. The absorbance (450 nm) was measured in a BioTek ELx808 plate reader (BioTek Instruments, Inc., Winooski, VT), and the concentrations (in nanograms per milliliter) were calculated using known standards.

### Pol II recruitment assay.

Thioglycolate-elicited mouse macrophages were pretreated with medium alone or medium plus 100 U/ml recombinant mouse IFN-β (PBL) for 3 h. Subsequently, 100 ng/ml P3C or 100 ng/ml LPS was added for an additional 3 h. RNA polymerase II recruitment to the CXCL1 promoter was determined by a chromatin immunoprecipitation (ChIP) assay using a ChIP IT Expresses-Enzymatic kit from Active Motif according to the manufacturer’s instructions. Briefly, macrophages were fixed and DNA was fragmented by enzymatic digestion. RNA Pol II immunoprecipitation was carried out overnight at 4°C using a monoclonal antibody directed against mouse RNA Pol II (Active Motif). Precipitated CXCL1 DNA fragments were quantified by PCR using primers amplifying a region of the CXCL1 promoter region: CCTCTTCACATGCCTCCCTG (forward) and CGGGGATGGAAGCTTGTCTT (reverse). Changes in CXCL1 were normalized to the changes in the control gene actin by use of the primers CCTCTGGGTGTGGATGTCAC (forward) and TGTCCATTCAATCCAGGCCC (reverse).

### Statistics.

Statistically significant differences between two groups were determined using an unpaired, two-tailed Student's *t* test, with significance set at a *P* value of <0.05. For comparisons between ≥3 groups, a one-way analysis of variance, followed by Tukey’s multiple-comparison test, was carried out, with significance determined to be a *P* value of <0.05. For survival studies, a log-rank (Mantel-Cox) test was used. All analyses were carried out using GraphPad Prism (v.7) software.

### Data availability.

The data that support the findings of this study are available upon request from the corresponding author.
