# Supplementary figures and images for: Influenza “Trains” the Host for Enhanced Susceptibility to Secondary Bacterial Infection
Source: mBio. 2019 May 7;10(3):e00810-19. doi: 10.1128/mBio.00810-19 (PMC6509193; doi:10.1128/mBio.00810-19)

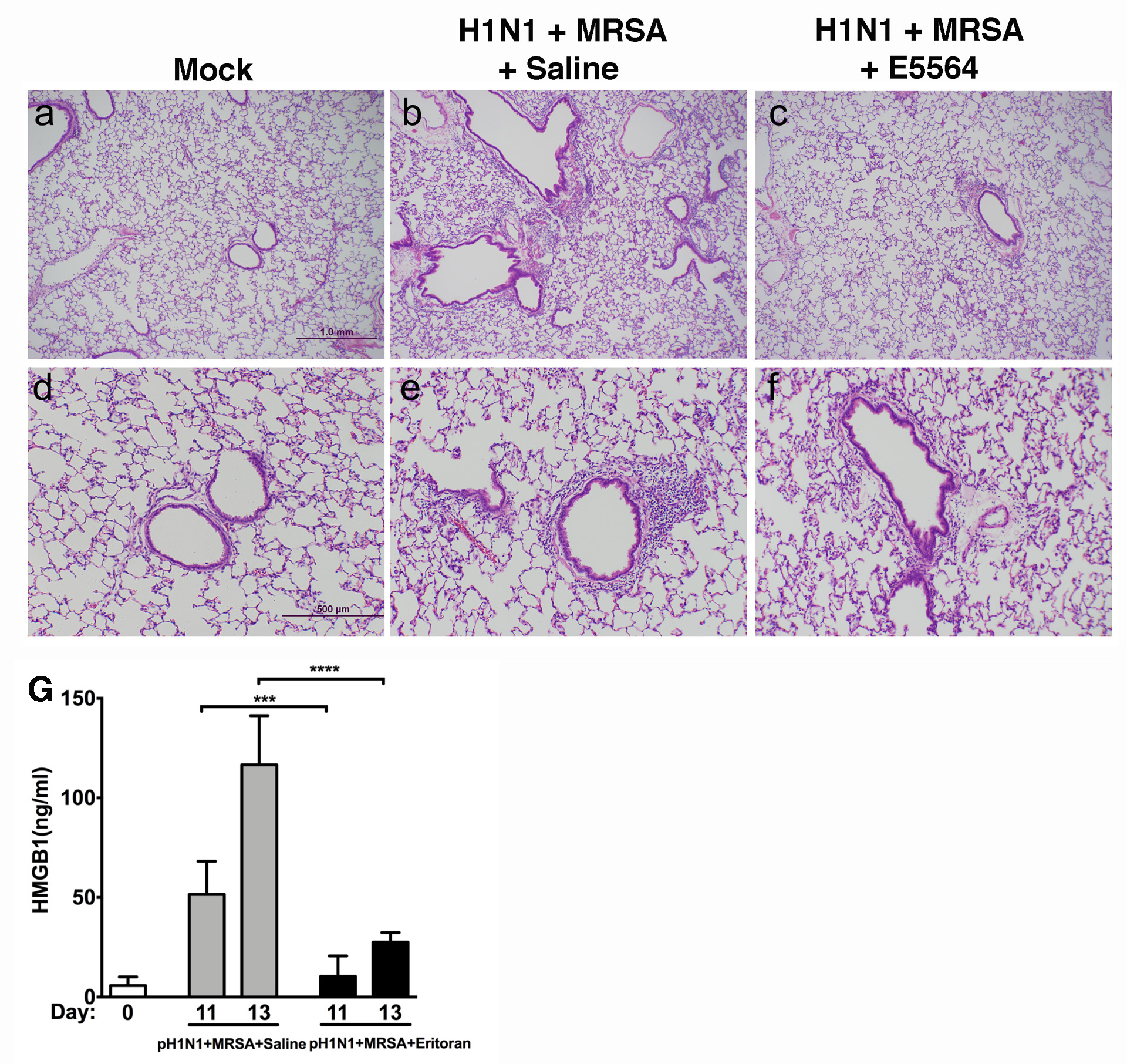

Supplement: FIG S1 [file mBio.00810-19-sf001.jpg]

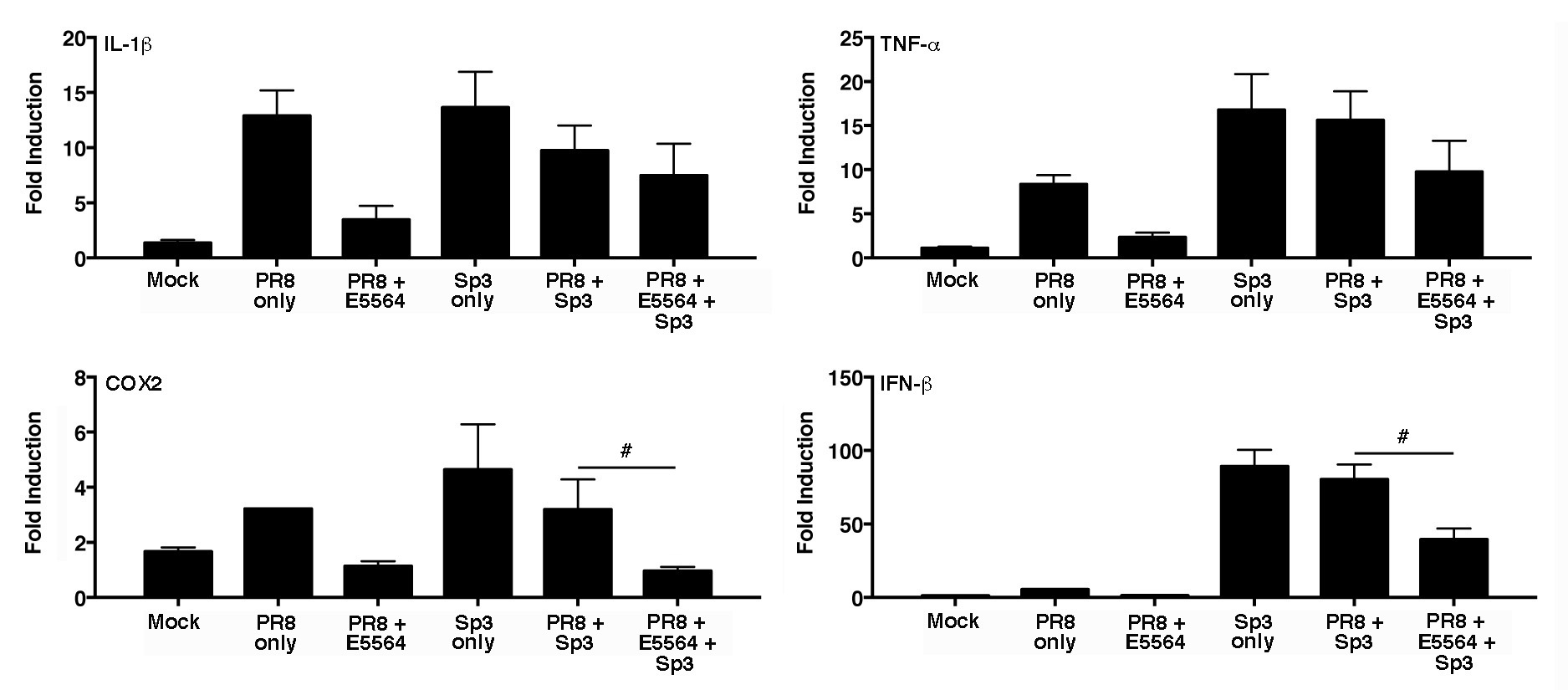

Supplement: FIG S2 [file mBio.00810-19-sf002.jpg]

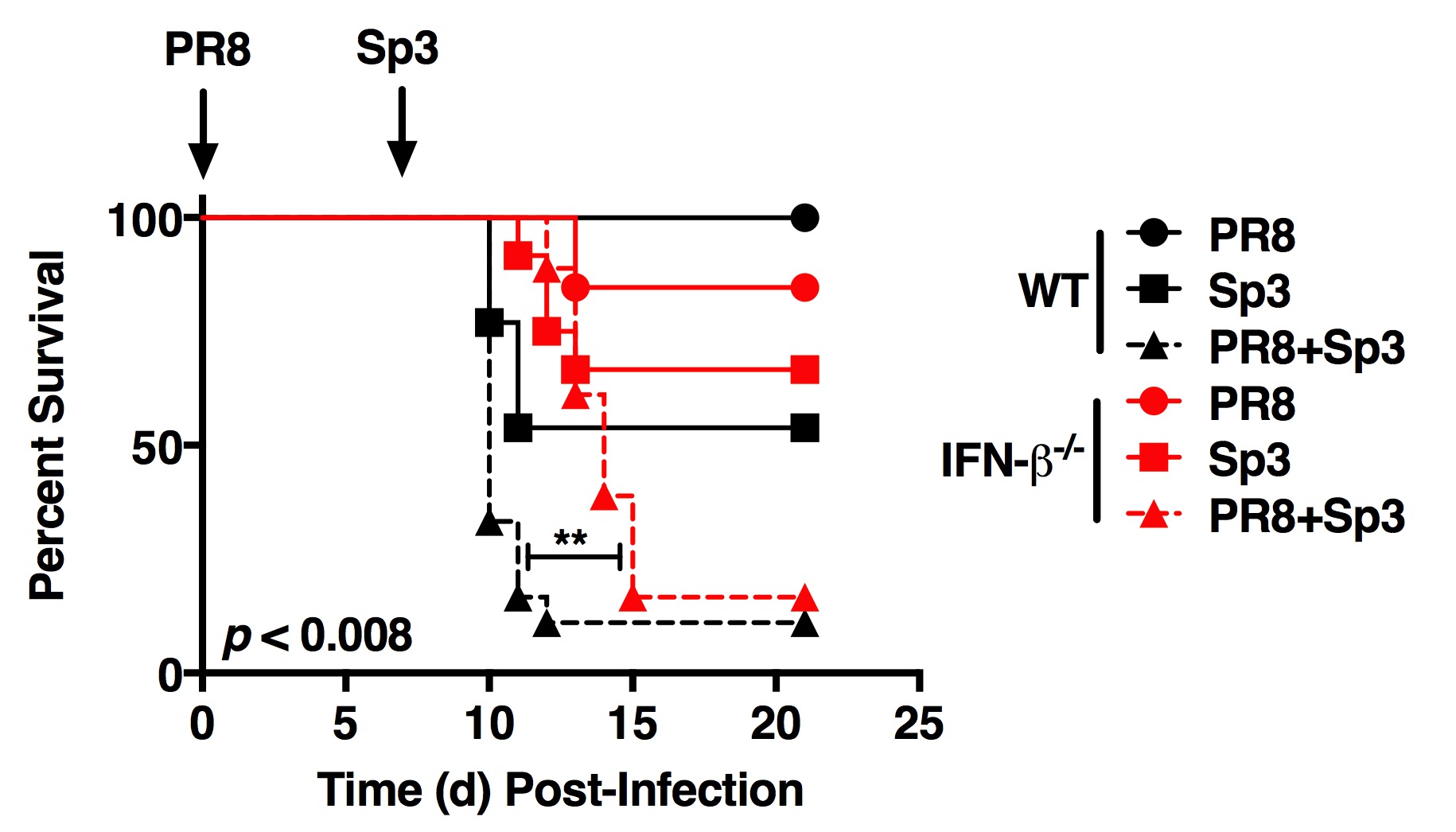

Supplement: FIG S3 [file mBio.00810-19-sf003.jpg]

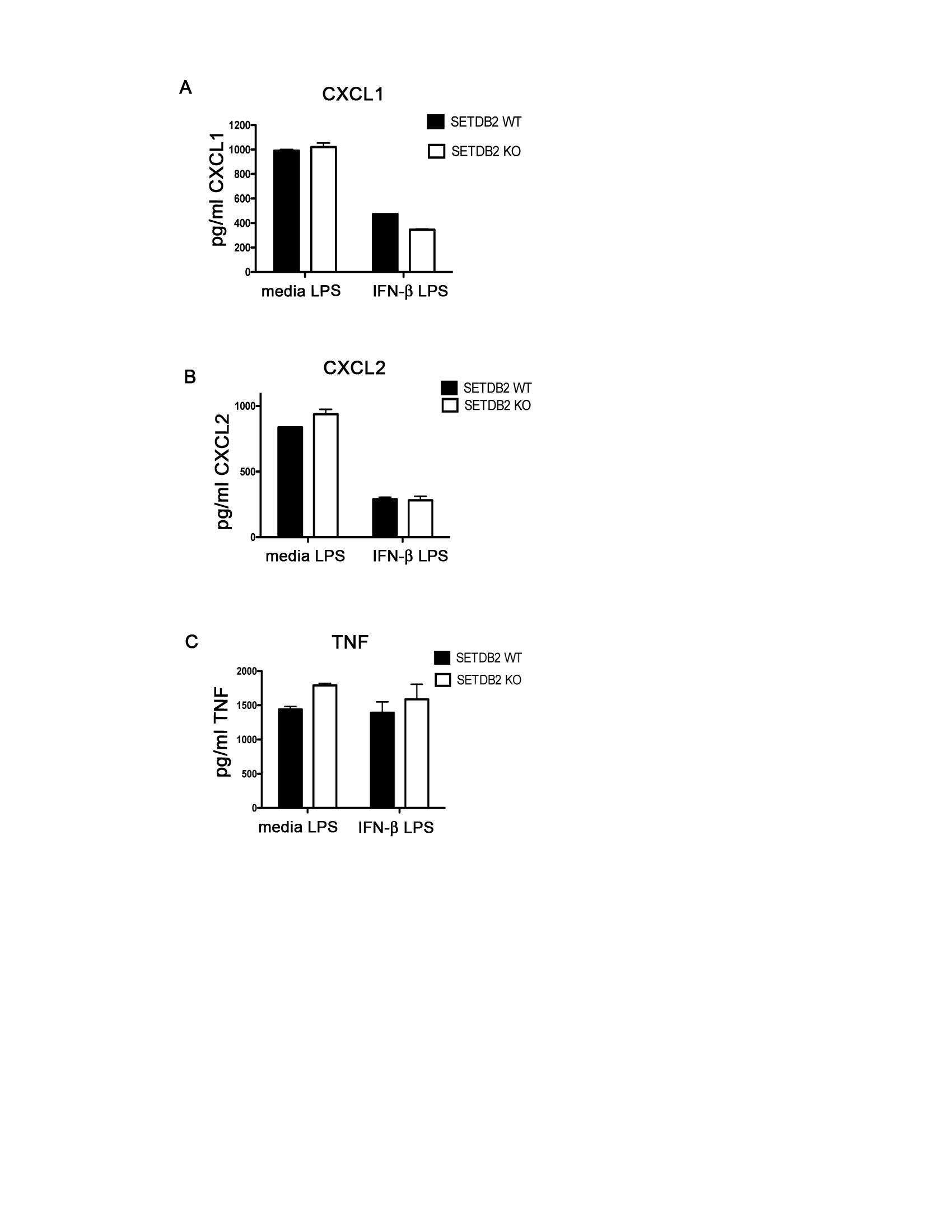

Supplement: FIG S4 [file mBio.00810-19-sf004.jpg]
